# Ceramide and ceramide 1-phosphate in health and disease

**DOI:** 10.1186/1476-511X-9-15

**Published:** 2010-02-05

**Authors:** Lide Arana, Patricia Gangoiti, Alberto Ouro, Miguel Trueba, Antonio Gómez-Muñoz

**Affiliations:** 1 Department of Biochemistry and Molecular Biology. Faculty of Science and Technology. University of the Basque Country (UPV/EHU). P.O. Box 644. 48080 - Bilbao, Spain

## Abstract

Sphingolipids are essential components of cell membranes, and many of them regulate vital cell functions. In particular, ceramide plays crucial roles in cell signaling processes. Two major actions of ceramides are the promotion of cell cycle arrest and the induction of apoptosis. Phosphorylation of ceramide produces ceramide 1-phosphate (C1P), which has opposite effects to ceramide. C1P is mitogenic and has prosurvival properties. In addition, C1P is an important mediator of inflammatory responses, an action that takes place through stimulation of cytosolic phospholipase A2, and the subsequent release of arachidonic acid and prostaglandin formation. All of the former actions are thought to be mediated by intracellularly generated C1P. However, the recent observation that C1P stimulates macrophage chemotaxis implicates specific plasma membrane receptors that are coupled to Gi proteins. Hence, it can be concluded that C1P has dual actions in cells, as it can act as an intracellular second messenger to promote cell survival, or as an extracellular receptor agonist to stimulate cell migration.

## Introduction

Sphingolipids play essential roles in normal cell and tissue homeostasis as well as in the establishment and progression of numerous diseases. In particular, ceramide is the central core in sphingolipid metabolism, but has also been involved in the regulation of signal transduction processes. Specifically, ceramides induce cell cycle arrest and promote apoptosis, a form of programmed cell death [1,2]. Also, ceramides play important roles in the regulation of autophagy, cell differentiation, survival, and inflammatory responses [3-11], and have been associated with insulin resistance through activation of protein phosphatase 2A and the subsequent dephosphorylation and inactivation of Akt (also known as protein kinase B (PKB)) [12-14]. Cell ceramides typically have long *N*-acyl chains ranging from 16 to 26 carbons in length [15-17]. However, in many studies short-chain analogs (N-acetylsphingosine, or C_2_-ceramide, N-hexanoylsphingosine, or C6-ceramide, and N-octanoylsphingosine, or C8-ceramide) have been used in experiments because these are more water soluble than long-chain ceramides. Formation of ceramide is also relevant because it is the precursor of important bioactive sphingolipids that can also regulate cellular functions, as discussed below.

**Figure 1:**
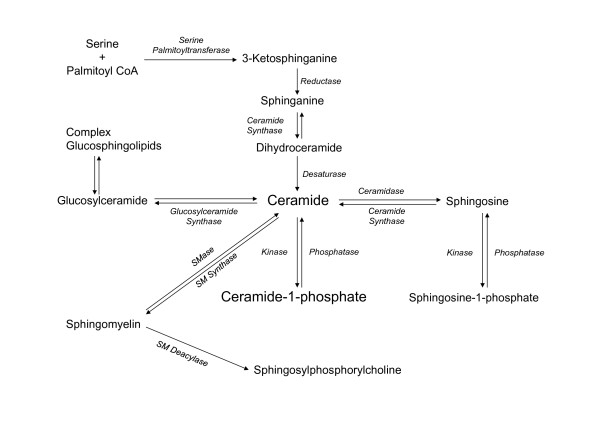
**Formation of bioactive sphingolipids in mammalian cells**. Ceramide can be produced by degradation of sphingomyelin (SM) by sphingomyelinases (SMase), or by *de novo *synthesis through the concerted action of serine palmitoyltransferase and dihydroceramide synthase. It can also be generated through metabolism of more complex sphingolipids. Ceramide can be metabolized to ceramide-1-phosphate by ceramide kinase, or to glucosylceramide by glucosylceramide synthase (GCS). The reverse reaction is catalyzed by ceramide-1-phosphate phosphatase, or by lipid phosphate phosphatases. Alternatively, ceramide can be degraded by ceramidases to form sphingosine, which can, in turn, be phosphorylated to sphingosine-1-phosphate by sphingosine kinase. The reverse reaction is catalyzed by sphingosine-1-phosphate phosphatases, or by lipid phosphate phosphatases. Sphingomyelin *N*-deacylase generates sphingosylphosphorylcholine.

A major metabolite of ceramide is ceramide-1-phosphate (C1P), which is generated through direct phosphorylation of ceramide by ceramide kinase (CerK) (Fig. 1). There is increasing evidence suggesting that C1P can regulate cell proliferation and apoptosis [7,18], and Chalfant and co-workers have elegantly demonstrated that C1P is a potent pro-inflammatory agent (Reviewed in [19,20]). In addition, C1P plays an important role in phagocytosis [21,22], and we have recently demonstrated that is a key factor in the regulation of macrophage chemotaxis. The aim of the present review is to discuss recent progress in C1P biology with especial emphasis in the context of health and disease.

## Synthesis of Bioactive Sphingolipids

Although sphingosine is the simplest sphingolipid, ceramide is considered to be the central structure in sphingolipid metabolism. Fig. 1 shows that ceramide can be generated by three major mechanisms: 1) the *de novo *synthesis pathway is an anabolic route that begins with condensation of the amino acid serine and palmitoyl-CoA to form 3-ketosphinganine in a reaction that is catalyzed by serine palmitoyltransferase (SPT); reduction of 3-ketosphinganine to sphinganine follows immediately; acylation of sphinganine by dihydroceramide synthase (CerS, also known as Lass) then generates dihydroceramide. The last step of this pathway is catalyzed by a desaturase through introduction of a trans-4, 5 double bond in the dihydroceramide molecule to yield ceramide (Fig. 1). Concerning CerS six different genes have been identified in mammalian cells. Interestingly, the different CerS isoforms produce ceramide with different N-acyl chains. The reason why there are so many of these genes when most of the other enzymes in the sphingolipid biosynthetic pathway exist in only one or two isoforms is not known. However, it is possible that ceramides containing different fatty acids play different roles in cell biology (reviewed in [23]). For details on SPT and CerS activities the reader is referred to other excellent reviews by Hannun and Obeid [2,5,24], and Merrill and co-workers [11,25]. Also, very elegant reviews by Kolesnick et al. [26], Goñi and Alonso [27], and Cremesti et al. [28] specifically address the important roles of SMase activities, enzymology, and compartmentalization in cell biology. Once synthesized, ceramide can be used for synthesis of complex sphingolipids, through intervention of different biosynthetic enzymes, including glucosyl or galactosyl ceramide synthases to form cerebrosides or gangliosides, or it can incorporate a phosphocholine head group from phosphatidylcholine (PC) to form SM through the action of SM synthases. Formation of glucosylceramide is particularly important because of its role in conferring drug resistance to tumor cells [29]. In addition, ceramide can be directly phosphorylated by ceramide kinase (CerK) to form C1P (Fig. 1), which is a key regulator of cell homeostasis [18,30] and has been implicated in inflammatory responses [19,20,31]. 2) The second major mechanism for ceramide generation is a catabolic pathway involving activation of SMases to form phosphorylcholine and ceramide directly (Fig. 1). There are three distinct forms of SMases in mammalian cells that can be discriminated *in vitro *by their optima pH: acid, neutral and alkaline SMases. Whilst acid SMase and neutral SMase are involved in signal transduction processes, the alkaline form of SMase is responsible for digestion of dietary SM in the intestine. The alkaline SMase isoform has now been re-named NPP7 because of its similarity to the nucleotide-pyrophosphatase/phosphodiesterase (NPP) family of enzymes. In addition to its role in SM digestion, a potential implication of this enzyme in cell signaling processes has also been suggested [32]. In particular alkaline SMase has been shown to inhibit cell proliferation in HT-29 colon carcinoma cells [33]. 3) The third important mechanism for generating ceramide is the sphingosine salvage pathway, in which sphingosine (produced from the metabolism of complex sphingolipids) is re-cycled to ceramide through the action of CerS. As mentioned above, another important enzyme that can control the levels of ceramide is sphingomyelin synthase (SMS) because it catalyzes the transfer of phosphorylcholine from phosphatidylcholine (PC) to ceramide, thereby releasing diacylglycerol (DAG) and lowering the levels of ceramide to produce SM. Interestingly, we have recently reported that SMS is implicated in the stimulation of PKC-α by C1P, an action that is linked to the mitogenic effect of this phosphosphingolipid in primary macrophages [34]. Ceramide can also be metabolized back to sphingosine by the action of specific ceramidases (Fig. 1). Sphingosine is also bioactive. It was first described as the physiological inhibitor of protein kinase C (PKC) [35]. There are many reports showing that PKC is inhibited by exogenous sphingosine, and Merrill and co-workers demonstrated that also endogenously generated sphingosine can inhibit protein kinase C very potently [36]. In turn, sphingosine can control the activity of other key enzymes involved in the regulation of metabolic or cell signaling pathways such as the Mg^2+ ^dependent form of phosphatidate phosphohydrolase [37,38], phospholipase D (PLD) [39], or diacylglycerol kinase (DAGK) [40,41] in a variety of cell types. In addition, sphingosine has been recently reported to be a ligand of the steroidogenic factor 1 (SF1) receptor, which is a nuclear receptor that plays a critical role in endocrine development of sex differentiation [42]. Endogenous sphingosine was found to be bound to this receptor under basal conditions, and treatment with cAMP decreased the amount of sphingosine bound to the receptor resulting in inhibition of cAMP-dependent CYP17 gene transcription [43]. Phosphorylation of sphingosine produces sphingosine 1-phosphate (S1P), which can regulate a variety of cellular functions including cell growth and survival, differentiation, and angiogenesis [19,44-46]. In addition, S1P stimulates cortisol and aldosterone secretion potently in cells of the zona fasciculata, and zona glomerulosa, respectively, suggesting that S1P is implicated in the regulation of steroidogenesis, and steroid hormone actions [47,48]. Two sphingosine kinases (SphKs) have so far been identified in mammalian cells, SphK1 and SphK2, which exhibit different biochemical properties and regulation. The roles of S1P and SphKs in cell biology have been extensively reviewed elsewhere [42,49].

### Ceramides

Besides its role as the precursor of complex sphingolipids ceramide is a signaling molecule capable of regulating vital cellular functions including apoptosis, cell growth, differentiation, senescence, diabetes, insulin resistance, inflammation, neurodegenerative disorders, or atherosclerosis[2-5,15,35,50-56]. In this connection, it should be pointed out that the topology of ceramide generation is crucial for determination of its functions as a bioregulatory molecule, with compartmentalization being essential for separation of signaling and metabolic pools within cells. Indeed, the enzymes that regulate ceramide metabolism show distinct subcellular localization and topology (reviewed in [2]). For instance, the plasma membrane of cells contains caveolae-associated neutral SMase, and a fraction of acid SMase, and the ceramides that are generated by these enzymes may have different functions. The enzymology, and compartmentalization of sphingomyelinases have been reviewed elsewhere [26-28]. Another important aspect of ceramide action concerns its transport from the ER, where it is synthesized, to the Golgi apparatus, the primary site of SM and glycosphingolipid synthesis. Hanada et al [57] recently demonstrated the existence of a specific protein that is involved in SM biosynthesis and acts as a ceramide transfer protein (CERT) in a non-vesicular manner. This protein has two domains involved in the transport of ceramide: one that recognizes ceramide and mediates its intermembrane transfer, termed the START domain, and a phosphatidylinositol binding domain (PH domain) with selectivity towards phosphatidylinositol-4-phosphate, a lipid that is enriched in the Golgi and that could serve as the site for ceramide delivery by CERT [57]. Ceramide generation at the plasma membrane exerts distinct and specific functions including aggregation of the Fas receptor, and effects on protein kinase C (PKC), but not other effects mediated by endogenous ceramides such as apoptosis, or cell cycle arrest [2]. Although the regulation of PKC activity by ceramides has already been reported, the results are still controversial. In this regard, ceramides have been shown to activate PKC-α and to inhibit PKC-α in renal mesangial cells [58]. They have also been shown to induce the translocation of PKC-α from the cytosol to the membrane [59], the translocation of PKC-δ and PKC-ε from the membrane to the cytosol [60], and the translocation of PKC-δ from the cytosol to the mitochondria [61]. Also, ceramide was shown to induce apoptosis by translocation, tyrosine phosphorylation and activation of PKC-δ in the Golgi complex [62]. Another important target of ceramide is phospholipase D (PLD), which is a key regulatory enzyme responsible for generation of phosphatidic acid (PA), a potent mitogenic agent, and a precursor of important second messengers including lysoPA and diacylglycerol (DAG) [7]. We first reported that the cell-permeable ceramides N-acetylsphingosine (C2-ceramide) and N-hexanoylsphingosine (C6-ceramide), or exogenous bacterial sphingomyelinase, which can generate ceramides at the plasma membrane of cells, inhibited agonist-stimulated PLD activity potently in intact rat fibroblasts [63,64] or macrophages [65-67]. PLD inhibition by ceramides has also been demonstrated in several other cell types [47,68-70], as well as in cell-free systems [71,72], or digitonin-permeabilized fibroblasts that were incubated with GTPγS [63]. However, the physiological significance of PLD inhibition by ceramides is still unclear.

Sphingolipids are also important because they are implicated in atherogenic processes (reviewed by Steinbrecher et al. [73]). In particular, ceramides, glycosphingolipids and S1P have been shown to accumulate in atherosclerotic lesions, and to participate in the regulation of signal transduction pathways that are implicated in atherogenesis. Ceramides and S1P can be generated by the action of oxidatively modified low density lipoproteins (LDL), or by pro-inflammatory cytokines. These bioactive sphingolipids can upregulate the expression of adhesion molecules and promote migration and adhesion of monocytes to the sites of lesions. In fact, early and intermediate atheromas are rich in macrophages and smooth muscle cells, and show evidence of active cell proliferation [74].

With regards to ceramide metabolism, the enzymes responsible for its degradation, have recently gained particular interest because of their involvement in various diseases. In particular ceramidases, would generate sphingosine directly, and sphingosine can be readily converted to S1P, a potent mitogenic agent and tumor promoter. Details on protein sequence, chromosomal location, tissue distribution, and subcellular localizations of the different ceramidases have been recently reviewed by Mao and Obeid [75]. Ceramidases have been implicated in the mitogenic effect of oxidized LDL (oxLDL), probably by enhancing the production of S1P [76]. Also, dysregulation of mesangial cell proliferation or death involves altered ceramidase activities [77-79] supporting a role of this enzyme in diabetic nephropathy. An involvement of the three different types of ceramidases (acid, neutral and alkaline) in the development of type 2 diabetes, insulin resistance and metabolic syndrome has also been reported [80-84]. Ceramidases appear to also be involved in some of the apoptotic effects promoted by nitric oxide [58,85-87] and inflammatory cytokines [88-99], the antiapoptotic properties of growth factors [100,101], and in the promotion of embryo survival by removing ceramides from newly formed embryos, thereby inhibiting the default apoptosis pathway [102]. Moreover, ceramidases attenuate peptidoglycan-induced COX-2 expression in macrophages [92], and the *P. aeruginosa *ceramidase enhances hemolysis induced phospholipase C [103]. Increasing evidence points to important roles of ceramidases, specially the *Asah1 *isoform, in the outcome and progression of cancer, and the response of tumors to therapy (reviewed in [33,95,104,105]. *Asah1 *is overexpressed in several cancer cell lines and cancer tissues [106-111], which appears to contribute to decreasing the levels of ceramide and increasing those of S1P. Multiple reports confirm the relationship between *Asah1 *activity and radio or chemotherapy resistance, as well as the interest of *Asah1 *inhibitors as anticancer drugs. Also, in most cases, Asah1 inhibition induces apoptosis. In fact, high levels of *Asah1 *expression were found in a radiation resistant glioblastoma cell line when exposed to gamma-radiation, and sensitivity to radiation was achieved by treatment with the ceramidase inhibitor *N*-oleoylethanolamine (NOE), which significantly increased ceramide levels, caspase activation and apoptosis [60]. In search for ceramidase inhibitors, most efforts have been directed to *Asah1 *inhibition, because of their potential used as antiproliferative and cytostatic drugs for cancer chemotherapy. Ceramidase inhibitors have also been used in models other than cancer. For example, incubation of smooth muscle cells with oxLDL increased the activities of both acid and alkaline ceramidases and the mitogenic effect of oxLDL was inhibited by DMAPP, suggesting a role for ceramidases (probably through formation of S1P) in the mitogenic effect of oxLDL [76].

### Ceramide 1-phosphate

Phosphorylation of ceramide seems to be the major mechanism for generation of C1P in cells. The only enzyme so far identified to induce the biosynthesis of C1P in mammalian cells is ceramide kinase (CerK). This enzyme was first observed in brain synaptic vesicles [112], and then found in human leukemia HL-60 monocytes [55]. CerK was found to be present in both the microsomal membrane fraction, and the cytosolic fraction of cells [113]. It was postulated that C1P traffics from the Golgi apparatus along the secretory pathway to the plasma membrane, and then released into the extracellular milieu to bind to acceptor proteins such as albumin or lipoproteins [114]. Recent work by Chalfant and co-workers [115] showed that CerK specifically utilizes ceramide transported to the trans-Golgi apparatus by ceramide transport protein (CERT). In fact, downregulation of CERT by RNA interference resulted in strong inhibition of newly synthesized C1P, suggesting that CERT plays a critical role in C1P formation. By contrast, Boath et al [114] recently reported that the transport of ceramides to the vicinity of CerK was not dependent on CERT. The reason for such discrepancy is unknown at present, but it might be possible that different cell types might have different subcellular distribution of CerK, and that expression of this enzyme might not be equal in all cell types. Concerning regulation of the enzyme activity, the dependency on Ca^2+ ^ions is well established. CerK was also proposed to be regulated by phosphorylation/dephosphorylation processes [116]. In addition, CerK location and activity seem to require the integrity of its PH domain, which includes a myristoylation site [116]. Another interesting aspect is that although CerK is the only enzyme so far described for generation of C1P in mammalian cells, bone marrow-derived macrophages (BMDM) from CerK-null mice (CerK-/-) still produced significant levels of C1P, suggesting the existence of a metabolic pathway, other than ceramide/CerK, for generation of C1P [114]. In particular, formation of C16-C1P, which is a major species of C1P in cells, was not abolished in (CerK-/-) BMDM. We have previously speculated that two alternative pathways for generation of C1P in cells might be the transfer of a long acyl-CoA chain to S1P by a putative acyl transferase, or cleavage of SM by a PLD-like activity, similar to the existing arthropod or bacterial SMase D. However, work from our own lab [117] and that of others [114] have shown that acylation of S1P to form C1P does not occur in mammalian cells. In addition, we found no evidence for intervention of SMase D activity when using rat fibroblasts. Nonetheless, these possibilities should be further explored in other cell types. Human CerK was cloned by Sugiura and co-workers [118]. The protein sequence has 537 amino acids with two protein sequence motifs, an N-terminus that encompasses a sequence motif known as a pleckstrin homology (PH) domain (amino acids 32-121), and a C-terminal region containing a Ca^2+^/calmodulin binding domain (amino acids 124-433). It was found that leucine 10 in the PH domain is essential for its catalytic activity [119]. Also, it was shown that interaction between the PH domain of CerK and phosphatidylinositol 4,5-bisphosphate regulates the plasma membrane targeting and C1P levels [120]. More recently, the existence of a conserved cysteine motif in CerK that is also essential for its function was reported [121]. Also, it has been suggested that subcellular localization of CerK requires the interplay of their PH domain-containing N-terminal regions together with the C-terminal domains [122]. Concerning substrate specificity, phosphorylation of ceramide by CerK is stereospecific [123]. It was reported that a minimum of a 12-carbon acyl chain is required for normal CerK activity, whereas the short-chain ceramide analogues C_8_-ceramide, C_4_-ceramide, or C_2_-ceramide were poor substrates for this enzyme. It was concluded that CerK phosphorylates only the naturally occurring D-erythro-ceramides [123]. However, Van Overloops and co-workers [124] observed that C_2_-ceramide is a good substrate for CerK, when albumin is used as a carrier, and that C_2_-ceramide can be converted to C_2_-C1P within cells. This raises the possibility that C_2_-C1P is also a natural sphingolipid, capable of eliciting important biologic effects, as previously demonstrated (i.e. stimulation of cell proliferation [125]). The importance of CerK in cell signaling was highlighted using specific RNAi to inhibit this enzyme activity. This treatment blocked arachidonic acid (AA) release and PGE_2 _production in response to ATP, the calcium ionophore A23187 and interleukin 1-β [19,126]. The relevance of this enzyme in cell biology was also highlighted in studies using CerK-/- mice; specifically, Bornancin and co-workers found a potent reduction in the amount of neutrophils in blood and spleen of these mice, whereas the amount of leukocytes, other than neutrophils, was increased in these animals. These observations pointed to an important role of CerK in neutrophil homeostasis [127]. Recently, a human ceramide kinase-like (CerKL) enzyme was identified in retina [128], and subsequently cloned [129]. However, this enzyme was unable to phosphorylate ceramide, or other related lipids, under conditions commonly used to measure CerK activity, and therefore its role in cell biology remains unclear. Importantly, intracellular formation of C1P was observed after challenging A549 lung adenocarcinoma cells with interleukin 1-β [126], and after treatment of bone marrow-derived macrophages with M-CSF [130]. Also of importance, C1P levels were substantially decreased in apoptotic macrophages, suggesting that C1P plays an important role in cell survival [18,117].

#### Role of ceramide 1-phosphate in cell growth and survival

We recently reported that the mechanisms by which C1P exerts its mitogenic effects involve stimulation of the mitogen-activated protein kinase kinase (MEK)/Extracellularly regulated kinases 1-2 (ERK1-2), phosphatidylinositol 3-kinase (PI3-K)/Akt (or PKB), and c-Jun terminal kinase (JNK) pathways [130]. We also found that C1P causes stimulation of the DNA binding activity of the transcription factor NF-κB, and increases the expression of glycogen synthase kinase-3β (GSK-3β) leading to up-regulation of cyclin D1 and c-Myc, which are important markers of cell proliferation. Moreover, we have evidence suggesting that C1P-stimulated macrophage proliferation, implicates activation of SMS as well as translocation and activation of PKC-α [34], and that phospholipase D (PLD), intracellular calcium levels, or cAMP are not involved in this process [125,131].

In addition to its mitogenic effect, we also observed that C1P is a potent inhibitor of apoptosis [117,132]. This finding was further supported by Mitra and co-workers [133] who found that down-regulation of CerK in mammalian cells reduced growth and promoted apoptosis in lung epithelial cells. However, Graf and co-workers reported that exogenous addition of the cell permeable C_2_-ceramide to cells overexpressing CerK led to C_2_-C1P formation and induction of apoptosis [134]. This contradictory observation can be explained by the fact that overexpression of CerK in the presence of abnormally high concentrations of ceramide (exogenously supplied) would cause and overwhelming increase in the intracellular levels of C1P, thereby reaching C1P concentrations that are toxic for cells. In fact, we observed that in contrast to relatively low concentrations of C1P, which stimulate cell growth and inhibit apoptosis, relatively higher concentrations of C1P are toxic and can kill the cells [117,125]. Concerning apoptosis, we also found that apoptotic bone marrow-derived macrophages have high acid SMase activity and high levels of ceramides compared to healthy cells [135,136]. Investigation into the mechanism whereby C1P exerts its anti-apoptotic effects led us to demonstrate that C1P caused potent inhibition of acid SMase and subsequent depletion of ceramide levels in intact macrophages [117]. C1P also blocked the activity of acid SMase in cell homogenates suggesting that inhibition of this enzyme occurs by direct physical interaction with C1P. It was concluded that C1P is a natural inhibitor of acid SMase, and that inhibition of this enzyme is a major mechanism whereby C1P promotes cell survival [117]. Also, this observation suggests that inhibition of acid SMase by C1P is not mediated through receptor interaction. Acid SMase was also inhibited by S1P in intact macrophages [136], but the mechanism by which this action is brought about remains to be established. Recent work from our lab [137] shows that ceramide levels are also increased in apoptotic alveolar NR8383 macrophages. However, contrary to bone marrow-derived macrophages, there was little activation of neutral and acid SMases in the alveolar macrophages, suggesting that ceramides were generated through a different pathway in these cells. Investigation into the mechanisms whereby ceramide levels increased in alveolar macrophages revealed that activation of SPT, which as mentioned above is the key regulatory enzyme of the de novo pathway of ceramide synthesis, was a major factor in this process. Like for SMases, inhibition of SPT activation by treatment with C1P substantially decreased ceramide generation, and prevented the macrophages from entering apoptosis. It was concluded that C1P promoted macrophage survival by blocking ceramide accumulation through inhibition of either SMase activity, or SPT, depending on cell type. The physiological relevance of the prosurvival effect of C1P was underscored by the demonstration that the intracellular levels of C1P were substantially decreased in apoptotic macrophages. It can be hypothesized that the decrease in C1P concentration could result in the release of acid SMase, or SPT, from inhibition, thereby triggering ceramide generation an apoptotic cell death.

A well-established mechanism by which growth factors promote cell survival is through activation of phosphatidylinositol 3-kinase (PI3-K), which can lead to stimulation of the transcription factor NF-κB, and expression of antiapoptotic genes. Using two different experimental approaches, we demonstrated that PI3-K was a target of C1P in bone marrow-derived macrophages [132]. PI3-K activation was demonstrated by immunoprecipitation of the enzyme from whole cell lysates and assayed *in vitro *using ^32^P-phosphatidylinositol. In addition, an *in vivo *approach provided evidence of phosphatidylinositol (3,4,5)-trisphosphate (PIP3) formation in intact cells that were prelabeled with ^32^P-orthophosphate [132]. Interestingly, PIP3, which is a major product of PI3-K activity, was shown to directly inhibit acid SMase [138]. Therefore, PI3-K activation may potentiate the inhibitory effect of C1P on acid SMase through generation of PIP3. We also observed that C1P stimulated phosphorylation of PKB, which is a target of kinases from different signaling pathways including PI3-K [139,140], cAMP or cAMP-dependent protein kinase (PKA) [141,142], and PKC-ζ [143]. C1P-induced phosphorylation of PKB was sensitive to inhibition by wortmannin or LY294002, which are selective inhibitors of PI3K. These two inhibitors also blocked the prosurvival effect of C1P indicating that PKB is downstream of PI3-K in macrophages, and important for the antiapoptotic effect of C1P [132]. C1P also caused IκB phosphorylation and stimulation of the DNA binding activity of NF-κB in primary cultures of mouse macrophages [132], and up-regulated the expression of anti-apoptotic Bcl-X_L_, which is a downstream target of NF-κB. The latter results provided the first evidence for a novel biological role of natural C1P in the regulation of cell survival by the PI3-K/PKB/NF-κB pathway in mammalian cells [132].

As mentioned above, C1P can be metabolized to ceramide by phosphatase activity, and then further converted to sphingosine and S1P by ceramidases and sphingosine kinases. Therefore, it could be speculated that the effects of C1P might be mediated through C1P-derived metabolites. However, ceramides and C1P are antagonistic signals, and C1P is unable to mimic many of the effects of sphingosine or S1P (i.e. PLD activation, adenylyl cyclase inhibition, or Ca^2+ ^mobilization) [7,64,125,131]. Also, ceramides can decrease the expression of Bcl-X_L _[19], whereas C1P causes its up-regulation [132]. Finally, no ceramidases capable of converting C1P to S1P have so far been identified, and S1P and C1P inhibit acid SMase by different mechanisms [117,136]. Therefore, it can be concluded that C1P acts on its own right to regulate cell functions. The above observations suggest that the activity of the enzymes involved in ceramide and C1P metabolism must be strictly regulated. Any alteration in the balance between ceramides and C1P could potentially result in metabolic dysfunctions, and could be fatal for cells.

#### Ceramide 1-phosphate and the control of inflammatory responses

C1P has been demonstrated to be proinflammatory, which in principle is beneficial for protecting the organism against infection or injury. Inflammatory mediators include chemokynes, cytokines, vasoactive amines, products of proteolitic cascades, phospholipases, different forms of eicosanoids, and some sphingolipids. Generation of proinflammatory metabolites, however, should be blocked or at least reduced when inflammation becomes out of control, so as to protect the organism from major damage. Concerning phospholipases, a key mediator of inflammatory responses is cytosolic PLA_2 _(cPLA_2_), an enzyme that has been involved in receptor-dependent and independent release of arachidonic acid and eicosanoid production. With regards to sphingolipids, some of them have also been described as important mediators of inflammatory responses. For instance, ceramide was initially described as pro-inflammatory for different cell types [144-147], and more recently it has been implicated in the development of allergic asthmatic responses and airway inflammation [148]. In addition, exogenous addition of the short-chain cell permeable C_2_-ceramide, to cultured astrocytes upregulated the expression of 12-lipoxygenase, thereby leading to generation of reactive oxygen species (ROS) and the initiation of inflammatory responses [149]. Acid sphingomyelinase-derived ceramide has also been involved in PAF-mediated pulmonary edema [150]. Subsequently, it was proposed that at least some of the pro-inflammatory effects of ceramides might in fact be mediated by its conversion to C1P. The first report on the regulation of arachidonic acid (AA) release and the production of prostaglandins by C1P was by Chalfant's group [126]. These authors demonstrated that C1P potently and specifically stimulated AA release and prostanoid synthesis in A549 lung adenocarcinoma cells. In the same report, the authors showed that C1P could be generated intracellularly through stimulation of CerK by the action of interleukin 1-β. In a later report, the same group demonstrated that the mechanism whereby C1P stimulates AA release occurs through direct activation of cPLA_2 _[151]. Subsequently, Subramanian and co-workers [152] found that C1P is a positive allosteric activator of group IV cPLA_2_, and that it enhances the interaction of the enzyme with phosphatidylcholine. The authors concluded that C1P may function to recruit cPLA_2_α to intracellular membranes and that it allosterically increases the catalytic ability of the membrane-associated enzyme [152]. In addition, recent studies demonstrated that activation of group IV cPLA_2 _by C1P is chain length-specific. In particular, C1P with acyl chains equal or higher than 6 carbons were able to efficiently activate cPLA_2_α in vitro, whereas shorter acyl chains (in particular C_2_-C1P) were unable to activate this enzyme. C1P was suggested to act in coordination with S1P to ensure maximal production of prostaglandins [153]. For details on the role of C1P in inflammatory response the reader is referred to elegant reviews by Lamour and Chalfant [115]; Wijesinghe et al [154] and Chalfant and Spiegel [19]. It should also be pointed out that C1P is involved in other inflammatory processes including stimulation of phagocytosis in neutrophils [21,22], activation of degranulation in mast cells [113], and more recently, stimulation of macrophage migration [155]. Nonetheless, apart from its clearly proinflammatory actions, C1P might act as antiinflammatory under specific conditions. In this context, it was postulated that activation of acid SMase plays an important role in pulmonary infections as it facilitates internalization of bacteria into lung epithelial cells [156]. Therefore, the recent finding that C1P potently inhibits acid SMase [116] could be important to reduce or prevent infection in the lung, an action that would obviously result in the inhibition of inflammatory responses.

#### Ceramide 1-phosphate mediates macrophage migration

Macrophages are involved in a number of chronic diseases that are characterized by unregulated chronic inflammation. These include autoimmune diseases, atherosclerosis, or multiple sclerosis [157], as well as tumor progression and metastasis [158]. Using Raw 264.7 macrophages, our group has recently demonstrated that exogenous addition of C1P potently stimulated cell migration [155]. This action could only be observed when C1P was applied exogenously, but not when C1P was generated intracellularly. The intracellular levels of C1P were enhanced using different experimental approaches, including agonist stimulation of CerK, or delivery of C1P using the photolabile caged-C1P compounds 7-(diethylamino)-coumarin (DECM), or 4-bromo-5-hydroxy-2-nitrobenzhydryl (BHNB) [159] to the cells in culture but macrophages failed to migrate (A. Ouro et al., unpublished work). These observations led to identify a specific plasma membrane receptor that stimulates chemotaxis upon ligation with C1P. This receptor had low affinity for C1P, with a K_d _value of approximately 7.8 μM. In addition, studies using GTPγS, and pertussis toxin, which potently blocks Gi proteins, provided evidence that the C1P receptor is coupled to a G_i _protein. Interestingly, ligation of the receptor with C1P caused potent phosphorylation of ERK1-2 and PKB, suggesting that these kinases are downstream of receptor activation. Of importance, inhibition of these pathways with selective inhibitors of MEK, the enzyme that phosphorylate ERK, and selective inhibitors of PI3-K, completely abolished C1P-stimulated macrophage migration. Furthermore, C1P stimulated the DNA binding activity of NF-κB, which is downstream of PKB or ERK, and blockade of this transcription factor also resulted in complete inhibition of macrophage migration. These observations suggested that MEK/ERK1-2, PI3-K/PKB (Akt) and NF-κB are crucial components of the cascade of events leading to stimulation of cell migration by C1P. It is possible that this newly identified receptor as well as the enzymes responsible for C1P generation might be important targets for treatment of illnesses that are associated to inflammation and cell migration, such as atherosclerosis or cancer. In this connection, two inhibitors of CerK have been recently described. One of these inhibitors is an analog of a previously reported SphK inhibitor named F-12509A [160], which inhibits CerK at μmolar concentrations without affecting the activities of SphK or diacylglycerol kinases. A second compound named NVP-231 (adamantane-1-carboxylic acid (2-benzoylamino-benzothiazol-6-yl) amide) [161], inhibited CerK potently in a competitive and reversible manner at low nanomolar concentrations. Interestingly, when NVP-231 was combined with tamoxifen, a drug that is commonly used for treatment of breast cancer [162,163], it synergistically increased ceramide levels and blocked cell growth [161]. Also of interest, recent work by Zor and co-workers has produced a C1P analogue named phosphoceramide analogue-1 (PCERA-1), which has potent anti-inflammatory properties [164]. The activity of PCERA-1 seems to be mediated by a cell membrane receptor that is distinct to the C1P receptor described here. PCERA-1, and perhaps other compounds that may be eventually derived from modification of its original structure, might turn to also be useful tools for developing alternative strategies for treatment of inflammatory diseases.

## Concluding Remarks

Detailed knowledge of the mechanisms controlling ceramide and C1P levels, including expression of the enzymes involved in their metabolism, and the receptors implicated in their actions, may be essential for developing molecular strategies to counteract metabolic disorders. Specifically serine palmitoyltransferases, ceramide synthases, sphingomyelinases, ceramide kinase, ceramidases, and the different sphingolipid receptors are likely to be major targets for controlling sphingolipid actions, and metabolism. Finding selective inhibitors of these enzymes, as well as agonists and antagonists of these receptors will enhance our knowledge and understanding on how these molecules can control physiological and pathological processes including cell growth, differentiation, migration, neurodegeneration, cell death, inflammation, and cancer.

## Abbreviations

BMDM: bone marrow-derived macrophages; C_2_-ceramide: *N*-acetylsphingosine; C8-ceramide: *N*-octanoylsphingosine; C1P: ceramide-1-phosphate; DAG: diacylglycerol; ERK: extracellular regulated kinase; MAPK: mitogen-activated protein kinase; M-CSF: monocyte-colony stimulating factor; OxLDL: oxidized low density lipoproteins; LPP: lipid phosphate phosphatase; PA: phosphatidate; PC: phosphatidylcholine; PE: phosphatidylethanolamine; PI: phosphatidylinositol; PI3-K: phosphatidylinositol 3-kinase; PIP3: phosphatidylinositol (3,4,5) trisphosphate; PS: phosphatidylserine; PLA_2_: phospholipase A_2_; PKB: protein kinase B; PKC: protein kinase C; PLC: phospholipase C; PLD: phospholipase D; SM: sphingomyelin; S1P: sphingosine-1-phosphate; SphK: sphingosine kinase.

## Competing interests

The authors declare that they have no competing interests.

## Authors' contributions

All authors participated in the writing of the manuscript.

All authors read and approved the final manuscript.

## References

[B1] Zheng W Kollmeyer J Symolon H Momin A Munter E Wang E Kelly S Allegood JC Liu Y Peng Q Ceramides and other bioactive sphingolipid backbones in health and disease: lipidomic analysis, metabolism and roles in membrane structure, dynamics, signaling and autophagy Biochim Biophys Acta 2006 1758 1864 1884 10.1016/j.bbamem.2006.08.009 17052686

[B2] Hannun YA Obeid LM The Ceramide-centric universe of lipid-mediated cell regulation: stress encounters of the lipid kind J Biol Chem 2002 277 25847 25850 10.1074/jbc.R200008200 12011103

[B3] Kolesnick R Golde DW The sphingomyelin pathway in tumor necrosis factor and interleukin-1 signaling Cell 1994 77 325 328 10.1016/0092-8674(94)90147-3 8181053

[B4] Hannun YA The sphingomyelin cycle and the second messenger function of ceramide J Biol Chem 1994 269 3125 3128 8106344

[B5] Hannun YA Obeid LM Ceramide: an intracellular signal for apoptosis Trends Biochem Sci 1995 20 73 77 10.1016/S0968-0004(00)88961-6 7701566

[B6] Dressler KA Mathias S Kolesnick RN Tumor necrosis factor-alpha activates the sphingomyelin signal transduction pathway in a cell-free system Science 1992 255 1715 1718 10.1126/science.1313189 1313189

[B7] Gomez-Munoz A Modulation of cell signalling by ceramides Biochim Biophys Acta 1998 1391 92 109 9518566 10.1016/s0005-2760(97)00201-4

[B8] Mathias S Dressler KA Kolesnick RN Characterization of a ceramide-activated protein kinase: stimulation by tumor necrosis factor alpha Proc Natl Acad Sci USA 1991 88 10009 10013 10.1073/pnas.88.22.10009 1946418 PMC52856

[B9] Mathias S Kolesnick R Ceramide: a novel second messenger Adv Lipid Res 1993 25 65 90 8368154

[B10] Okazaki T Bielawska A Bell RM Hannun YA Role of ceramide as a lipid mediator of 1 alpha,25-dihydroxyvitamin D3-induced HL-60 cell differentiation J Biol Chem 1990 265 15823 15831 2394750

[B11] Menaldino DS Bushnev A Sun A Liotta DC Symolon H Desai K Dillehay DL Peng Q Wang E Allegood J Sphingoid bases and de novo ceramide synthesis: enzymes involved, pharmacology and mechanisms of action Pharmacol Res 2003 47 373 381 10.1016/S1043-6618(03)00054-9 12676511

[B12] Adams JM Pratipanawatr T Berria R Wang E DeFronzo RA Sullards MC Mandarino LJ Ceramide content is increased in skeletal muscle from obese insulin-resistant humans Diabetes 2004 53 25 31 10.2337/diabetes.53.1.25 14693694

[B13] Schmitz-Peiffer C Protein kinase C and lipid-induced insulin resistance in skeletal muscle Ann NY Acad Sci 2002 967 146 157 12079844 10.1111/j.1749-6632.2002.tb04272.x

[B14] Stratford S Hoehn KL Liu F Summers SA Regulation of insulin action by ceramide: dual mechanisms linking ceramide accumulation to the inhibition of Akt/protein kinase B J Biol Chem 2004 279 36608 36615 10.1074/jbc.M406499200 15220355

[B15] Merrill AH Jr De novo sphingolipid biosynthesis: a necessary, but dangerous, pathway J Biol Chem 2002 277 25843 25846 10.1074/jbc.R200009200 12011104

[B16] Merrill AH Jr Sullards MC Allegood JC Kelly S Wang E Sphingolipidomics: high-throughput, structure-specific, and quantitative analysis of sphingolipids by liquid chromatography tandem mass spectrometry Methods 2005 36 207 224 10.1016/j.ymeth.2005.01.009 15894491

[B17] Pettus BJ Bielawska A Kroesen BJ Moeller PD Szulc ZM Hannun YA Busman M Observation of different ceramide species from crude cellular extracts by normal-phase high-performance liquid chromatography coupled to atmospheric pressure chemical ionization mass spectrometry Rapid Commun Mass Spectrom 2003 17 1203 1211 10.1002/rcm.1043 12772277

[B18] Gomez-Munoz A Ceramide-1-phosphate: a novel regulator of cell activation FEBS Lett 2004 562 5 10 10.1016/S0014-5793(04)00211-X 15069950

[B19] Chalfant CE Spiegel S Sphingosine 1-phosphate and ceramide 1-phosphate: expanding roles in cell signaling J Cell Sci 2005 118 4605 4612 10.1242/jcs.02637 16219683

[B20] Lamour NF Chalfant CE Ceramide-1-phosphate: the "missing" link in eicosanoid biosynthesis and inflammation Mol Interv 2005 5 358 367 10.1124/mi.5.6.8 16394251

[B21] Hinkovska-Galcheva V Boxer LA Kindzelskii A Hiraoka M Abe A Goparju S Spiegel S Petty HR Shayman JA Ceramide 1-phosphate, a mediator of phagocytosis J Biol Chem 2005 280 26612 26621 10.1074/jbc.M501359200 15899891

[B22] Hinkovska-Galcheva VT Boxer LA Mansfield PJ Harsh D Blackwood A Shayman JA The formation of ceramide-1-phosphate during neutrophil phagocytosis and its role in liposome fusion J Biol Chem 1998 273 33203 33209 10.1074/jbc.273.50.33203 9837889

[B23] Pewzner-Jung Y Ben-Dor S Futerman AH When do Lasses (longevity assurance genes) become CerS (ceramide synthases)?: Insights into the regulation of ceramide synthesis J Biol Chem 2006 281 25001 25005 10.1074/jbc.R600010200 16793762

[B24] Hannun YA Obeid LM Principles of bioactive lipid signalling: lessons from sphingolipids Nat Rev Mol Cell Biol 2008 9 139 150 10.1038/nrm2329 18216770

[B25] Desai K Sullards MC Allegood J Wang E Schmelz EM Hartl M Humpf HU Liotta DC Peng Q Merrill AH Jr Fumonisins and fumonisin analogs as inhibitors of ceramide synthase and inducers of apoptosis Biochim Biophys Acta 2002 1585 188 192 12531553 10.1016/s1388-1981(02)00340-2

[B26] Kolesnick RN Goni FM Alonso A Compartmentalization of ceramide signaling: physical foundations and biological effects J Cell Physiol 2000 184 285 300 10.1002/1097-4652(200009)184:3<285::AID-JCP2>3.0.CO;2-3 10911359

[B27] Goni FM Alonso A Sphingomyelinases: enzymology and membrane activity FEBS Lett 2002 531 38 46 10.1016/S0014-5793(02)03482-8 12401200

[B28] Cremesti AE Goni FM Kolesnick R Role of sphingomyelinase and ceramide in modulating rafts: do biophysical properties determine biologic outcome? FEBS Lett 2002 531 47 53 10.1016/S0014-5793(02)03489-0 12401201

[B29] Gouaze-Andersson V Cabot MC Glycosphingolipids and drug resistance Biochim Biophys Acta 2006 1758 2096 2103 10.1016/j.bbamem.2006.08.012 17010304

[B30] Gomez-Munoz A Ceramide 1-phosphate/ceramide, a switch between life and death Biochim Biophys Acta 2006 1758 2049 2056 10.1016/j.bbamem.2006.05.011 16808893

[B31] Pettus BJ Chalfant CE Hannun YA Sphingolipids in inflammation: roles and implications Curr Mol Med 2004 4 405 418 10.2174/1566524043360573 15354871

[B32] Hertervig E Nilsson A Cheng Y Duan RD Purified intestinal alkaline sphingomyelinase inhibits proliferation without inducing apoptosis in HT-29 colon carcinoma cells J Cancer Res Clin Oncol 2003 129 577 582 10.1007/s00432-003-0466-2 12920578 PMC12161909

[B33] Duan RD Nilsson A Metabolism of sphingolipids in the gut and its relation to inflammation and cancer development Prog Lipid Res 2009 48 62 72 10.1016/j.plipres.2008.04.003 19027789

[B34] Gangoiti P Granado MH Arana L Ouro A Gomez-Munoz A Activation of protein kinase C-alpha is essential for stimulation of cell proliferation by ceramide 1-phosphate FEBS Lett 2010 584 3 517 24 Epub 2009 Nov 27 10.1016/j.febslet.2009.11.086 19948174

[B35] Hannun YA Loomis CR Merrill AH Jr Bell RM Sphingosine inhibition of protein kinase C activity and of phorbol dibutyrate binding in vitro and in human platelets J Biol Chem 1986 261 12604 12609 3462188

[B36] Smith ER Jones PL Boss JM Merrill AH Jr Changing J774A.1 cells to new medium perturbs multiple signaling pathways, including the modulation of protein kinase C by endogenous sphingoid bases J Biol Chem 1997 272 5640 5646 10.1074/jbc.272.9.5640 9038174

[B37] Gomez-Munoz A Hamza EH Brindley DN Effects of sphingosine, albumin and unsaturated fatty acids on the activation and translocation of phosphatidate phosphohydrolases in rat hepatocytes Biochim Biophys Acta 1992 1127 49 56 1320939 10.1016/0005-2760(92)90200-f

[B38] Jamal Z Martin A Gomez-Munoz A Brindley DN Plasma membrane fractions from rat liver contain a phosphatidate phosphohydrolase distinct from that in the endoplasmic reticulum and cytosol J Biol Chem 1991 266 2988 2996 1993672

[B39] Natarajan V Jayaram HN Scribner WM Garcia JG Activation of endothelial cell phospholipase D by sphingosine and sphingosine-1-phosphate Am J Respir Cell Mol Biol 1994 11 221 229 8049083 10.1165/ajrcmb.11.2.8049083

[B40] Sakane F Yamada K Kanoh H Different effects of sphingosine, R59022 and anionic amphiphiles on two diacylglycerol kinase isozymes purified from porcine thymus cytosol FEBS Lett 1989 255 409 413 10.1016/0014-5793(89)81134-2 2551742

[B41] Yamada K Sakane F Imai S Takemura H Sphingosine activates cellular diacylglycerol kinase in intact Jurkat cells, a human T-cell line Biochim Biophys Acta 1993 1169 217 224 7548113

[B42] Liu H Sugiura M Nava VE Edsall LC Kono K Poulton S Milstien S Kohama T Spiegel S Molecular cloning and functional characterization of a novel mammalian sphingosine kinase type 2 isoform J Biol Chem 2000 275 19513 19520 10.1074/jbc.M002759200 10751414

[B43] Urs AN Dammer E Sewer MB Sphingosine regulates the transcription of CYP17 by binding to steroidogenic factor-1 Endocrinology 2006 147 5249 5258 10.1210/en.2006-0355 16887917

[B44] Spiegel S English D Milstien S Sphingosine 1-phosphate signaling: providing cells with a sense of direction Trends Cell Biol 2002 12 236 242 10.1016/S0962-8924(02)02277-8 12062172

[B45] Spiegel S Milstien S Sphingosine-1-phosphate: an enigmatic signalling lipid Nat Rev Mol Cell Biol 2003 4 397 407 10.1038/nrm1103 12728273

[B46] Spiegel S Milstien S Sphingosine 1-phosphate, a key cell signaling molecule J Biol Chem 2002 277 25851 25854 10.1074/jbc.R200007200 12011102

[B47] Rabano M Pena A Brizuela L Marino A Macarulla JM Trueba M Gomez-Munoz A Sphingosine-1-phosphate stimulates cortisol secretion FEBS Lett 2003 535 101 105 10.1016/S0014-5793(02)03882-6 12560086

[B48] Brizuela L Rabano M Pena A Gangoiti P Macarulla JM Trueba M Gomez-Munoz A Sphingosine 1-phosphate: a novel stimulator of aldosterone secretion J Lipid Res 2006 47 1238 1249 10.1194/jlr.M500510-JLR200 16554657

[B49] Taha TA Hannun YA Obeid LM Sphingosine kinase: biochemical and cellular regulation and role in disease J Biochem Mol Biol 2006 39 113 131 16584625 10.5483/bmbrep.2006.39.2.113

[B50] Merrill AH Jr Cell regulation by sphingosine and more complex sphingolipids J Bioenerg Biomembr 1991 23 83 104 2010436 10.1007/BF00768840

[B51] Merrill AH Jr Jones DD An update of the enzymology and regulation of sphingomyelin metabolism Biochim Biophys Acta 1990 1044 1 12 2187537 10.1016/0005-2760(90)90211-f

[B52] Merrill AH Jr Schmelz EM Dillehay DL Spiegel S Shayman JA Schroeder JJ Riley RT Voss KA Wang E Sphingolipids--the enigmatic lipid class: biochemistry, physiology, and pathophysiology Toxicol Appl Pharmacol 1997 142 208 225 10.1006/taap.1996.8029 9007051

[B53] Kolesnick R The therapeutic potential of modulating the ceramide/sphingomyelin pathway J Clin Invest 2002 110 3 8 12093880 10.1172/JCI16127 PMC151041

[B54] Kolesnick RN 1,2-Diacylglycerols but not phorbol esters stimulate sphingomyelin hydrolysis in GH3 pituitary cells J Biol Chem 1987 262 16759 16762 3479432

[B55] Kolesnick RN Hemer MR Characterization of a ceramide kinase activity from human leukemia (HL-60) cells. Separation from diacylglycerol kinase activity J Biol Chem 1990 265 18803 18808 2172234

[B56] Hannun YA Functions of ceramide in coordinating cellular responses to stress Science 1996 274 1855 1859 10.1126/science.274.5294.1855 8943189

[B57] Hanada K Kumagai K Yasuda S Miura Y Kawano M Fukasawa M Nishijima M Molecular machinery for non-vesicular trafficking of ceramide Nature 2003 426 803 809 10.1038/nature02188 14685229

[B58] Huwiler A Fabbro D Pfeilschifter J Selective ceramide binding to protein kinase C-alpha and -delta isoenzymes in renal mesangial cells Biochemistry 1998 37 14556 14562 10.1021/bi981401i 9772184

[B59] Ibitayo AI Sladick J Tuteja S Louis-Jacques O Yamada H Groblewski G Welsh M Bitar KN HSP27 in signal transduction and association with contractile proteins in smooth muscle cells Am J Physiol 1999 277 G445 454 10444459 10.1152/ajpgi.1999.277.2.G445

[B60] Sawai H Okazaki T Takeda Y Tashima M Sawada H Okuma M Kishi S Umehara H Domae N Ceramide-induced translocation of protein kinase C-delta and -epsilon to the cytosol. Implications in apoptosis J Biol Chem 1997 272 2452 2458 10.1074/jbc.272.4.2452 8999958

[B61] Sumitomo M Ohba M Asakuma J Asano T Kuroki T Asano T Hayakawa M Protein kinase Cdelta amplifies ceramide formation via mitochondrial signaling in prostate cancer cells J Clin Invest 2002 109 827 836 11901191 10.1172/JCI14146 PMC150911

[B62] Kajimoto T Shirai Y Sakai N Yamamoto T Matsuzaki H Kikkawa U Saito N Ceramide-induced apoptosis by translocation, phosphorylation, and activation of protein kinase Cdelta in the Golgi complex J Biol Chem 2004 279 12668 12676 10.1074/jbc.M312350200 14715667

[B63] Gomez-Munoz A Martin A O'Brien L Brindley DN Cell-permeable ceramides inhibit the stimulation of DNA synthesis and phospholipase D activity by phosphatidate and lysophosphatidate in rat fibroblasts J Biol Chem 1994 269 8937 8943 8132631

[B64] Gomez-Munoz A Waggoner DW O'Brien L Brindley DN Interaction of ceramides, sphingosine, and sphingosine 1-phosphate in regulating DNA synthesis and phospholipase D activity J Biol Chem 1995 270 26318 26325 10.1074/jbc.270.44.26318 7592842

[B65] Gomez-Munoz A Martens JS Steinbrecher UP Stimulation of phospholipase D activity by oxidized LDL in mouse peritoneal macrophages Arterioscler Thromb Vasc Biol 2000 20 135 143 10634810 10.1161/01.atv.20.1.135

[B66] Gomez-Munoz A O'Brien L Hundal R Steinbrecher UP Lysophosphatidylcholine stimulates phospholipase D activity in mouse peritoneal macrophages J Lipid Res 1999 40 988 993 10357830

[B67] Gomez-Munoz A O'Brien L Steinbrecher UP The platelet-activating factor receptor antagonist L-659,989 inhibits phospholipase D activity Biochim Biophys Acta 1999 1438 247 252 10320807 10.1016/s1388-1981(99)00056-6

[B68] Rabano M Pena A Brizuela L Macarulla JM Gomez-Munoz A Trueba M Angiotensin II-stimulated cortisol secretion is mediated by phospholipase D Mol Cell Endocrinol 2004 222 9 20 10.1016/j.mce.2004.05.006 15249121

[B69] Venable ME Obeid LM Phospholipase D in cellular senescence Biochim Biophys Acta 1999 1439 291 298 10425402 10.1016/s1388-1981(99)00101-8

[B70] Perez-Andres E Fernandez-Rodriguez M Gonzalez M Zubiaga A Vallejo A Garcia I Matute C Pochet S Dehaye JP Trueba M Activation of phospholipase D-2 by P2X(7) agonists in rat submandibular gland acini J Lipid Res 2002 43 1244 1255 12177168

[B71] Abousalham A Liossis C O'Brien L Brindley DN Cell-permeable ceramides prevent the activation of phospholipase D by ADP-ribosylation factor and RhoA J Biol Chem 1997 272 1069 1075 10.1074/jbc.272.2.1069 8995404

[B72] Venable ME Bielawska A Obeid LM Ceramide inhibits phospholipase D in a cell-free system J Biol Chem 1996 271 24800 24805 10.1074/jbc.271.40.24800 8798752

[B73] Steinbrecher UP Gomez-Munoz A Duronio V Acid sphingomyelinase in macrophage apoptosis Curr Opin Lipidol 2004 15 531 537 10.1097/00041433-200410000-00006 15361788

[B74] Kockx MM De Meyer GR Buyssens N Knaapen MW Bult H Herman AG Cell composition, replication, and apoptosis in atherosclerotic plaques after 6 months of cholesterol withdrawal Circ Res 1998 83 378 387 9721694 10.1161/01.res.83.4.378

[B75] Mao C Obeid LM Ceramidases: regulators of cellular responses mediated by ceramide, sphingosine, and sphingosine-1-phosphate Biochim Biophys Acta 2008 1781 424 434 18619555 10.1016/j.bbalip.2008.06.002 PMC2614331

[B76] Auge N Nikolova-Karakashian M Carpentier S Parthasarathy S Negre-Salvayre A Salvayre R Merrill AH Jr Levade T Role of sphingosine 1-phosphate in the mitogenesis induced by oxidized low density lipoprotein in smooth muscle cells via activation of sphingomyelinase, ceramidase, and sphingosine kinase J Biol Chem 1999 274 21533 21538 10.1074/jbc.274.31.21533 10419457

[B77] Geoffroy K Troncy L Wiernsperger N Lagarde M El Bawab S Glomerular proliferation during early stages of diabetic nephropathy is associated with local increase of sphingosine-1-phosphate levels FEBS Lett 2005 579 1249 1254 10.1016/j.febslet.2004.12.094 15710421

[B78] Geoffroy K Wiernsperger N Lagarde M El Bawab S Bimodal effect of advanced glycation end products on mesangial cell proliferation is mediated by neutral ceramidase regulation and endogenous sphingolipids J Biol Chem 2004 279 34343 34352 10.1074/jbc.M403273200 15184394

[B79] Zager RA Conrad DS Burkhart K Ceramide accumulation during oxidant renal tubular injury: mechanisms and potential consequences J Am Soc Nephrol 1998 9 1670 1680 9727376 10.1681/ASN.V991670

[B80] Partovian C Ju R Zhuang ZW Martin KA Simons M Syndecan-4 regulates subcellular localization of mTOR Complex2 and Akt activation in a PKCalpha-dependent manner in endothelial cells Mol Cell 2008 32 140 149 10.1016/j.molcel.2008.09.010 18851840 PMC2578831

[B81] Straczkowski M Kowalska I Baranowski M Nikolajuk A Otziomek E Zabielski P Adamska A Blachnio A Gorski J Gorska M Increased skeletal muscle ceramide level in men at risk of developing type 2 diabetes Diabetologia 2007 50 2366 2373 10.1007/s00125-007-0781-2 17724577

[B82] Samad F Hester KD Yang G Hannun YA Bielawski J Altered adipose and plasma sphingolipid metabolism in obesity: a potential mechanism for cardiovascular and metabolic risk Diabetes 2006 55 2579 2587 10.2337/db06-0330 16936207

[B83] Blachnio-Zabielska A Zabielski P Baranowski M Gorski J Effects of Streptozotocin-induced Diabetes and Elevation of Plasma FFA on Ceramide Metabolism in Rat Skeletal Muscle Horm Metab Res 2010 42 1 7 10.1055/s-0029-1238322 19753513

[B84] Chavez JA Holland WL Bar J Sandhoff K Summers SA Acid ceramidase overexpression prevents the inhibitory effects of saturated fatty acids on insulin signaling J Biol Chem 2005 280 20148 20153 10.1074/jbc.M412769200 15774472

[B85] Franzen R Fabbro D Aschrafi A Pfeilschifter J Huwiler A Nitric oxide induces degradation of the neutral ceramidase in rat renal mesangial cells and is counterregulated by protein kinase C J Biol Chem 2002 277 46184 46190 10.1074/jbc.M204034200 12359735

[B86] Franzen R Pfeilschifter J Huwiler A Nitric oxide induces neutral ceramidase degradation by the ubiquitin/proteasome complex in renal mesangial cell cultures FEBS Lett 2002 532 441 444 10.1016/S0014-5793(02)03727-4 12482609

[B87] Pahan K Sheikh FG Khan M Namboodiri AM Singh I Sphingomyelinase and ceramide stimulate the expression of inducible nitric-oxide synthase in rat primary astrocytes J Biol Chem 1998 273 2591 2600 10.1074/jbc.273.5.2591 9446561

[B88] Amadou A Nawrocki A Best-Belpomme M Pavoine C Pecker F Arachidonic acid mediates dual effect of TNF-alpha on Ca2+ transients and contraction of adult rat cardiomyocytes Am J Physiol Cell Physiol 2002 282 C1339 1347 11997249 10.1152/ajpcell.00471.2001

[B89] Hatano Y Terashi H Arakawa S Katagiri K Interleukin-4 suppresses the enhancement of ceramide synthesis and cutaneous permeability barrier functions induced by tumor necrosis factor-alpha and interferon-gamma in human epidermis J Invest Dermatol 2005 124 786 792 10.1111/j.0022-202X.2005.23651.x 15816837

[B90] Radin MJ Holycross BJ Dumitrescu C Kelley R Altschuld RA Leptin modulates the negative inotropic effect of interleukin-1beta in cardiac myocytes Mol Cell Biochem 2008 315 179 184 10.1007/s11010-008-9805-6 18535786

[B91] Oral H Dorn GW Mann DL Sphingosine mediates the immediate negative inotropic effects of tumor necrosis factor-alpha in the adult mammalian cardiac myocyte J Biol Chem 1997 272 4836 4842 10.1074/jbc.272.8.4836 9030540

[B92] Lin CI Chen CN Chen JH Lee H Lysophospholipids increase IL-8 and MCP-1 expressions in human umbilical cord vein endothelial cells through an IL-1-dependent mechanism J Cell Biochem 2006 99 1216 1232 10.1002/jcb.20963 16795034

[B93] Kaszkin M Huwiler A Scholz K Bosch H van den Pfeilschifter J Negative regulation of interleukin-1beta-activated neutral sphingomyelinase by protein kinase C in rat mesangial cells FEBS Lett 1998 440 163 166 10.1016/S0014-5793(98)01445-8 9862447

[B94] Franzen R Pautz A Brautigam L Geisslinger G Pfeilschifter J Huwiler A Interleukin-1beta induces chronic activation and de novo synthesis of neutral ceramidase in renal mesangial cells J Biol Chem 2001 276 35382 35389 10.1074/jbc.M102153200 11457826

[B95] Zeidan YH Jenkins RW Korman JB Liu X Obeid LM Norris JS Hannun YA Molecular targeting of acid ceramidase: implications to cancer therapy Curr Drug Targets 2008 9 653 661 10.2174/138945008785132358 18691012 PMC3402562

[B96] Osawa Y Uchinami H Bielawski J Schwabe RF Hannun YA Brenner DA Roles for C16-ceramide and sphingosine 1-phosphate in regulating hepatocyte apoptosis in response to tumor necrosis factor-alpha J Biol Chem 2005 280 27879 27887 10.1074/jbc.M503002200 15946935

[B97] Strelow A Bernardo K Adam-Klages S Linke T Sandhoff K Kronke M Adam D Overexpression of acid ceramidase protects from tumor necrosis factor-induced cell death J Exp Med 2000 192 601 612 10.1084/jem.192.5.601 10974027 PMC2193270

[B98] De Vito WJ Xhaja K Stone S Prenatal alcohol exposure increases TNFalpha-induced cytotoxicity in primary astrocytes Alcohol 2000 21 63 71 10.1016/S0741-8329(00)00078-1 10946159

[B99] Nikolova-Karakashian M Morgan ET Alexander C Liotta DC Merrill AH Jr Bimodal regulation of ceramidase by interleukin-1beta. Implications for the regulation of cytochrome p450 2C11 J Biol Chem 1997 272 18718 18724 10.1074/jbc.272.30.18718 9228043

[B100] Coroneos E Martinez M McKenna S Kester M Differential regulation of sphingomyelinase and ceramidase activities by growth factors and cytokines. Implications for cellular proliferation and differentiation J Biol Chem 1995 270 23305 23309 10.1074/jbc.270.40.23305 7559485

[B101] Payne SG Brindley DN Guilbert LJ Epidermal growth factor inhibits ceramide-induced apoptosis and lowers ceramide levels in primary placental trophoblasts J Cell Physiol 1999 180 263 270 10.1002/(SICI)1097-4652(199908)180:2<263::AID-JCP14>3.0.CO;2-H 10395296

[B102] Eliyahu E Park JH Shtraizent N He X Schuchman EH Acid ceramidase is a novel factor required for early embryo survival FASEB J 2007 21 1403 1409 10.1096/fj.06-7016com 17264167

[B103] Okino N Ito M Ceramidase enhances phospholipase C-induced hemolysis by Pseudomonas aeruginosa J Biol Chem 2007 282 6021 6030 10.1074/jbc.M603088200 17202150

[B104] Liu X Elojeimy S Turner LS Mahdy AE Zeidan YH Bielawska A Bielawski J Dong JY El-Zawahry AM Guo GW Acid ceramidase inhibition: a novel target for cancer therapy Front Biosci 2008 13 2293 2298 10.2741/2843 17981711

[B105] Park JH Schuchman EH Acid ceramidase and human disease Biochim Biophys Acta 2006 1758 2133 2138 10.1016/j.bbamem.2006.08.019 17064658

[B106] Elojeimy S Liu X McKillop JC El-Zawahry AM Holman DH Cheng JY Meacham WD Mahdy AE Saad AF Turner LS Role of acid ceramidase in resistance to FasL: therapeutic approaches based on acid ceramidase inhibitors and FasL gene therapy Mol Ther 2007 15 1259 1263 10.1038/sj.mt.6300167 17426710

[B107] Seelan RS Qian C Yokomizo A Bostwick DG Smith DI Liu W Human acid ceramidase is overexpressed but not mutated in prostate cancer Genes Chromosomes Cancer 2000 29 137 146 10.1002/1098-2264(2000)9999:9999<::AID-GCC1018>3.0.CO;2-E 10959093

[B108] Perry DK Carton J Shah AK Meredith F Uhlinger DJ Hannun YA Serine palmitoyltransferase regulates de novo ceramide generation during etoposide-induced apoptosis J Biol Chem 2000 275 9078 9084 10.1074/jbc.275.12.9078 10722759

[B109] Ruckhaberle E Rody A Engels K Gaetje R von Minckwitz G Schiffmann S Grosch S Geisslinger G Holtrich U Karn T Kaufmann M Microarray analysis of altered sphingolipid metabolism reveals prognostic significance of sphingosine kinase 1 in breast cancer Breast Cancer Res Treat 2008 112 41 52 10.1007/s10549-007-9836-9 18058224

[B110] Ruckhaberle E Holtrich U Engels K Hanker L Gatje R Metzler D Karn T Kaufmann M Rody A Acid ceramidase 1 expression correlates with a better prognosis in ER-positive breast cancer Climacteric 2009 1 12 10.1080/13697130902939913 19905902

[B111] Saad AF Meacham WD Bai A Anelli V Elojeimy S Mahdy AE Turner LS Cheng J Bielawska A Bielawski J The functional effects of acid ceramidase overexpression in prostate cancer progression and resistance to chemotherapy Cancer Biol Ther 2007 6 1455 1460 17881906 10.4161/cbt.6.9.4623

[B112] Bajjalieh SM Martin TF Floor E Synaptic vesicle ceramide kinase. A calcium-stimulated lipid kinase that co-purifies with brain synaptic vesicles J Biol Chem 1989 264 14354 14360 2547795

[B113] Mitsutake S Kim TJ Inagaki Y Kato M Yamashita T Igarashi Y Ceramide kinase is a mediator of calcium-dependent degranulation in mast cells J Biol Chem 2004 279 17570 17577 10.1074/jbc.M312885200 14769792

[B114] Boath A Graf C Lidome E Ullrich T Nussbaumer P Bornancin F Regulation and traffic of ceramide 1-phosphate produced by ceramide kinase: comparative analysis to glucosylceramide and sphingomyelin J Biol Chem 2008 283 8517 8526 10.1074/jbc.M707107200 18086664

[B115] Lamour NF Stahelin RV Wijesinghe DS Maceyka M Wang E Allegood JC Merrill AH Jr Cho W Chalfant CE Ceramide kinase uses ceramide provided by ceramide transport protein: localization to organelles of eicosanoid synthesis J Lipid Res 2007 48 1293 1304 10.1194/jlr.M700083-JLR200 17392267

[B116] Baumruker T Bornancin F Billich A The role of sphingosine and ceramide kinases in inflammatory responses Immunol Lett 2005 96 175 185 10.1016/j.imlet.2004.09.001 15585321

[B117] Gomez-Munoz A Kong JY Salh B Steinbrecher UP Ceramide-1-phosphate blocks apoptosis through inhibition of acid sphingomyelinase in macrophages J Lipid Res 2004 45 99 105 10.1194/jlr.M300158-JLR200 14523050

[B118] Sugiura M Kono K Liu H Shimizugawa T Minekura H Spiegel S Kohama T Ceramide kinase, a novel lipid kinase. Molecular cloning and functional characterization J Biol Chem 2002 277 23294 23300 10.1074/jbc.M201535200 11956206

[B119] Kim TJ Mitsutake S Kato M Igarashi Y The leucine 10 residue in the pleckstrin homology domain of ceramide kinase is crucial for its catalytic activity FEBS Lett 2005 579 4383 4388 10.1016/j.febslet.2005.06.079 16081073

[B120] Kim TJ Mitsutake S Igarashi Y The interaction between the pleckstrin homology domain of ceramide kinase and phosphatidylinositol 4,5-bisphosphate regulates the plasma membrane targeting and ceramide 1-phosphate levels Biochem Biophys Res Commun 2006 342 611 617 10.1016/j.bbrc.2006.01.170 16488390

[B121] Lidome E Graf C Jaritz M Schanzer A Rovina P Nikolay R Bornancin F A conserved cysteine motif essential for ceramide kinase function Biochimie 2008 90 1560 1565 10.1016/j.biochi.2008.07.001 18662741

[B122] Rovina P Schanzer A Graf C Mechtcheriakova D Jaritz M Bornancin F Subcellular localization of ceramide kinase and ceramide kinase-like protein requires interplay of their Pleckstrin Homology domain-containing N-terminal regions together with C-terminal domains Biochim Biophys Acta 2009 1791 1023 1030 19501188 10.1016/j.bbalip.2009.05.009

[B123] Wijesinghe DS Massiello A Subramanian P Szulc Z Bielawska A Chalfant CE Substrate specificity of human ceramide kinase J Lipid Res 2005 46 2706 2716 10.1194/jlr.M500313-JLR200 16170208

[B124] Van Overloop H Denizot Y Baes M Van Veldhoven PP On the presence of C2-ceramide in mammalian tissues: possible relationship to etherphospholipids and phosphorylation by ceramide kinase Biol Chem 2007 388 315 324 10.1515/BC.2007.035 17338639

[B125] Gomez-Munoz A Duffy PA Martin A O'Brien L Byun HS Bittman R Brindley DN Short-chain ceramide-1-phosphates are novel stimulators of DNA synthesis and cell division: antagonism by cell-permeable ceramides Mol Pharmacol 1995 47 833 839 7746276

[B126] Pettus BJ Bielawska A Spiegel S Roddy P Hannun YA Chalfant CE Ceramide kinase mediates cytokine- and calcium ionophore-induced arachidonic acid release J Biol Chem 2003 278 38206 38213 10.1074/jbc.M304816200 12855693

[B127] Tuson M Marfany G Gonzalez-Duarte R Mutation of CERKL, a novel human ceramide kinase gene, causes autosomal recessive retinitis pigmentosa (RP26) Am J Hum Genet 2004 74 128 138 10.1086/381055 14681825 PMC1181900

[B128] Graf C Zemann B Rovina P Urtz N Schanzer A Reuschel R Mechtcheriakova D Muller M Fischer E Reichel C Neutropenia with Impaired Immune Response to Streptococcus pneumoniae in Ceramide Kinase-Deficient Mice J Immunol 2008 180 3457 3466 18292572 10.4049/jimmunol.180.5.3457

[B129] Bornancin F Mechtcheriakova D Stora S Graf C Wlachos A Devay P Urtz N Baumruker T Billich A Characterization of a ceramide kinase-like protein Biochim Biophys Acta 2005 1687 31 43 15708351 10.1016/j.bbalip.2004.11.012

[B130] Gangoiti P Granado MH Wang SW Kong JY Steinbrecher UP Gomez-Munoz A Ceramide 1-phosphate stimulates macrophage proliferation through activation of the PI3-kinase/PKB, JNK and ERK1/2 pathways Cell Signal 2008 20 726 736 10.1016/j.cellsig.2007.12.008 18234473

[B131] Gomez-Munoz A Frago LM Alvarez L Varela-Nieto I Stimulation of DNA synthesis by natural ceramide 1-phosphate Biochem J 1997 325 Pt 2 435 440 9230125 10.1042/bj3250435 PMC1218579

[B132] Gomez-Munoz A Kong JY Parhar K Wang SW Gangoiti P Gonzalez M Eivemark S Salh B Duronio V Steinbrecher UP Ceramide-1-phosphate promotes cell survival through activation of the phosphatidylinositol 3-kinase/protein kinase B pathway FEBS Lett 2005 579 3744 3750 10.1016/j.febslet.2005.05.067 15978590

[B133] Mitra P Maceyka M Payne SG Lamour N Milstien S Chalfant CE Spiegel S Ceramide kinase regulates growth and survival of A549 human lung adenocarcinoma cells FEBS Lett 2007 581 735 740 10.1016/j.febslet.2007.01.041 17274985 PMC2001272

[B134] Graf C Rovina P Tauzin L Schanzer A Bornancin F Enhanced ceramide-induced apoptosis in ceramide kinase overexpressing cells Biochem Biophys Res Commun 2007 354 309 314 10.1016/j.bbrc.2006.12.208 17222802

[B135] Hundal RS Gomez-Munoz A Kong JY Salh BS Marotta A Duronio V Steinbrecher UP Oxidized low density lipoprotein inhibits macrophage apoptosis by blocking ceramide generation, thereby maintaining protein kinase B activation and Bcl-XL levels J Biol Chem 2003 278 24399 24408 10.1074/jbc.M209179200 12750385

[B136] Gomez-Munoz A Kong J Salh B Steinbrecher UP Sphingosine-1-phosphate inhibits acid sphingomyelinase and blocks apoptosis in macrophages FEBS Lett 2003 539 56 60 10.1016/S0014-5793(03)00197-2 12650926

[B137] Granado MH Gangoiti P Ouro A Arana L Gomez-Munoz A Ceramide 1-phosphate inhibits serine palmitoyltransferase and blocks apoptosis in alveolar macrophages Biochim Biophys Acta 2009 1791 263 272 19416641 10.1016/j.bbalip.2009.01.023

[B138] Testai FD Landek MA Goswami R Ahmed M Dawson G Acid sphingomyelinase and inhibition by phosphate ion: role of inhibition by phosphatidyl-myo-inositol 3,4,5-triphosphate in oligodendrocyte cell signaling J Neurochem 2004 89 636 644 10.1046/j.1471-4159.2004.02374.x 15086520

[B139] Scheid MP Woodgett JR Unravelling the activation mechanisms of protein kinase B/Akt FEBS Lett 2003 546 108 112 10.1016/S0014-5793(03)00562-3 12829245

[B140] Scheid MP Huber M Damen JE Hughes M Kang V Neilsen P Prestwich GD Krystal G Duronio V Phosphatidylinositol (3,4,5)P3 is essential but not sufficient for protein kinase B (PKB) activation; phosphatidylinositol (3,4)P2 is required for PKB phosphorylation at Ser-473: studies using cells from SH2-containing inositol-5-phosphatase knockout mice J Biol Chem 2002 277 9027 9035 10.1074/jbc.M106755200 11781306

[B141] Sable CL Filippa N Hemmings B Van Obberghen E cAMP stimulates protein kinase B in a Wortmannin-insensitive manner FEBS Lett 1997 409 253 257 10.1016/S0014-5793(97)00518-8 9202156

[B142] Filippa N Sable CL Filloux C Hemmings B Van Obberghen E Mechanism of protein kinase B activation by cyclic AMP-dependent protein kinase Mol Cell Biol 1999 19 4989 5000 10373549 10.1128/mcb.19.7.4989 PMC84322

[B143] Van Kolen K Gilany K Moens L Esmans EL Slegers H P2Y12 receptor signalling towards PKB proceeds through IGF-I receptor cross-talk and requires activation of Src, Pyk2 and Rap1 Cell Signal 2006 18 1169 1181 10.1016/j.cellsig.2005.09.005 16236484

[B144] Serhan CN Haeggstrom JZ Leslie CC Lipid mediator networks in cell signaling: update and impact of cytokines Faseb J 1996 10 1147 1158 8751717 10.1096/fasebj.10.10.8751717

[B145] Manna SK Aggarwal BB IL-13 suppresses TNF-induced activation of nuclear factor-kappa B, activation protein-1, and apoptosis J Immunol 1998 161 2863 2872 9743347

[B146] Newton R Hart L Chung KF Barnes PJ Ceramide induction of COX-2 and PGE(2) in pulmonary A549 cells does not involve activation of NF-kappaB Biochem Biophys Res Commun 2000 277 675 679 10.1006/bbrc.2000.3722 11062012

[B147] Hayakawa M Jayadev S Tsujimoto M Hannun YA Ito F Role of ceramide in stimulation of the transcription of cytosolic phospholipase A2 and cyclooxygenase 2 Biochem Biophys Res Commun 1996 220 681 686 10.1006/bbrc.1996.0464 8607825

[B148] Masini E Giannini L Nistri S Cinci L Mastroianni R Xu W Comhair SA Li D Cuzzocrea S Matuschak GM Salvemini D Ceramide: a key signaling molecule in a Guinea pig model of allergic asthmatic response and airway inflammation J Pharmacol Exp Ther 2008 324 548 557 10.1124/jpet.107.131565 18042827

[B149] Prasad VV Nithipatikom K Harder DR Ceramide elevates 12-hydroxyeicosatetraenoic acid levels and upregulates 12-lipoxygenase in rat primary hippocampal cell cultures containing predominantly astrocytes Neurochem Int 2008 53 220 229 10.1016/j.neuint.2008.07.002 18680775

[B150] Goggel R Winoto-Morbach S Vielhaber G Imai Y Lindner K Brade L Brade H Ehlers S Slutsky AS Schutze S PAF-mediated pulmonary edema: a new role for acid sphingomyelinase and ceramide Nat Med 2004 10 155 160 10.1038/nm977 14704790

[B151] Pettus BJ Bielawska A Subramanian P Wijesinghe DS Maceyka M Leslie CC Evans JH Freiberg J Roddy P Hannun YA Chalfant CE Ceramide 1-phosphate is a direct activator of cytosolic phospholipase A2 J Biol Chem 2004 279 11320 11326 10.1074/jbc.M309262200 14676210

[B152] Subramanian P Stahelin RV Szulc Z Bielawska A Cho W Chalfant CE Ceramide 1-phosphate acts as a positive allosteric activator of group IVA cytosolic phospholipase A2 alpha and enhances the interaction of the enzyme with phosphatidylcholine J Biol Chem 2005 280 17601 17607 10.1074/jbc.M414173200 15743759

[B153] Pettus BJ Kitatani K Chalfant CE Taha TA Kawamori T Bielawski J Obeid LM Hannun YA The coordination of prostaglandin E2 production by sphingosine-1-phosphate and ceramide-1-phosphate Mol Pharmacol 2005 68 330 335 15900018 10.1124/mol.104.008722

[B154] Wijesinghe DS Lamour NF Gomez-Munoz A Chalfant CE Ceramide kinase and ceramide-1-phosphate Methods Enzymol 2007 434 265 292 full_text 17954253 10.1016/S0076-6879(07)34015-9

[B155] Granado MH Gangoiti P Ouro A Arana L Gonzalez M Trueba M Gomez-Munoz A Ceramide 1-phosphate (C1P) promotes cell migration Involvement of a specific C1P receptor Cell Signal 2009 21 405 412 10.1016/j.cellsig.2008.11.003 19041940

[B156] Gulbins E Kolesnick R Raft ceramide in molecular medicine Oncogene 2003 22 7070 7077 10.1038/sj.onc.1207146 14557812

[B157] Hendriks JJ Teunissen CE de Vries HE Dijkstra CD Macrophages and neurodegeneration Brain Res Brain Res Rev 2005 48 185 195 10.1016/j.brainresrev.2004.12.008 15850657

[B158] Condeelis J Pollard JW Macrophages: obligate partners for tumor cell migration, invasion, and metastasis Cell 2006 124 263 266 10.1016/j.cell.2006.01.007 16439202

[B159] Lankalapalli RS Ouro A Arana L Gomez-Munoz A Bittman R Caged ceramide 1-phosphate analogues: synthesis and properties J Org Chem 2009 74 8844 8847 10.1021/jo902076w 19908915 PMC2782442

[B160] Kim JW Inagaki Y Mitsutake S Maezawa N Katsumura S Ryu YW Park CS Taniguchi M Igarashi Y Suppression of mast cell degranulation by a novel ceramide kinase inhibitor, the F-12509A olefin isomer K1 Biochim Biophys Acta 2005 1738 82 90 16352467 10.1016/j.bbalip.2005.10.007

[B161] Graf C Klumpp M Habig M Rovina P Billich A Baumruker T Oberhauser B Bornancin F Targeting ceramide metabolism with a potent and specific ceramide kinase inhibitor Mol Pharmacol 2008 74 925 932 10.1124/mol.108.048652 18612076

[B162] Lavie Y Cao H Volner A Lucci A Han TY Geffen V Giuliano AE Cabot MC Agents that reverse multidrug resistance, tamoxifen, verapamil, and cyclosporin A, block glycosphingolipid metabolism by inhibiting ceramide glycosylation in human cancer cells J Biol Chem 1997 272 1682 1687 10.1074/jbc.272.3.1682 8999846

[B163] Cabot MC Giuliano AE Volner A Han TY Tamoxifen retards glycosphingolipid metabolism in human cancer cells FEBS Lett 1996 394 129 131 10.1016/0014-5793(96)00942-8 8843149

[B164] Goldsmith M Avni D Levy-Rimler G Mashiach R Ernst O Levi M Webb B Meijler MM Gray NS Rosen H Zor T A ceramide-1-phosphate analogue, PCERA-1, simultaneously suppresses tumour necrosis factor-alpha and induces interleukin-10 production in activated macrophages Immunology 2008 10.1111/j.1365-2567.2008.02928.x PMC2678186 18793216

